# DEB-TACE combined with ici for advanced squamous cell carcinoma of the skin: a case report

**DOI:** 10.3389/fonc.2025.1529976

**Published:** 2025-04-22

**Authors:** Zhuo Wu, Xuezhe Piao, Dongxu Kang, Lan Jin, Qiang Xu, Dehao Jin, Longzhen Piao

**Affiliations:** ^1^ Department of Oncology, Yanbian University Hospital, Yanji, Jilin, China; ^2^ Department of Intervention, Yanbian University Hospital, Yanji, Jilin, China

**Keywords:** DEB-TACE, ICI, CSCC, complete remission, case report

## Abstract

We report the case of an older female patient with squamous cell carcinoma of the skin, cTXN1M0, who initially presented with a mass of approximately 4.5 cm in diameter in the left temporal region, which had a tendency to break and bleed. Given the patient’s poor general status and ECOG score of grade 2, it was considered that she could not tolerate systemic chemotherapy. Therefore, we applied drug-eluting beads transarterial chemoembolization combined with immune checkpoint inhibitors to treat this patient and unexpectedly found that this regimen resulted in complete classified remission and in partial pathological response without significant adverse events.

## 1 Introduction

Cutaneous squamous cell carcinoma (cSCC) is one of the most common non-melanoma skin cancers, and its incidence has gradually increased in recent years (1). This form of cancer is predominantly found in exposed areas of the head and neck, most commonly in middle-aged and older adults, and has an approximately 5% chance of metastasizing to the parotid glands and cervical lymph nodes. Notably, the mortality rate increases significantly after metastasis. The known treatment regimens for cSCC include surgical treatment, chemotherapy, local non-surgical treatment, and radiotherapy. However, it should be noted that the above treatments have certain limitations (2). Currently, transarterial chemoembolization with drug-eluting beads (DEB-TACE) is commonly used for the local treatment of malignant tumors such as liver cancer and lung cancer. Through continuous exploration and practice, it has also been increasingly applied to the treatment of malignant tumors in other parts of the body (3). A study has indicated that DEB-TACE shows good therapeutic effects in the treatment of advanced head and neck cancer (4). After treatment, the local progression-free survival of patients can reach up to 13.6 months. Here, we report a case of DEB-TACE combined with immune checkpoint inhibitors (ICI) for the treatment of a patient with advanced head and neck cSCC, with remarkable efficacy.

## 2 Case presentation

**Figure 1:**
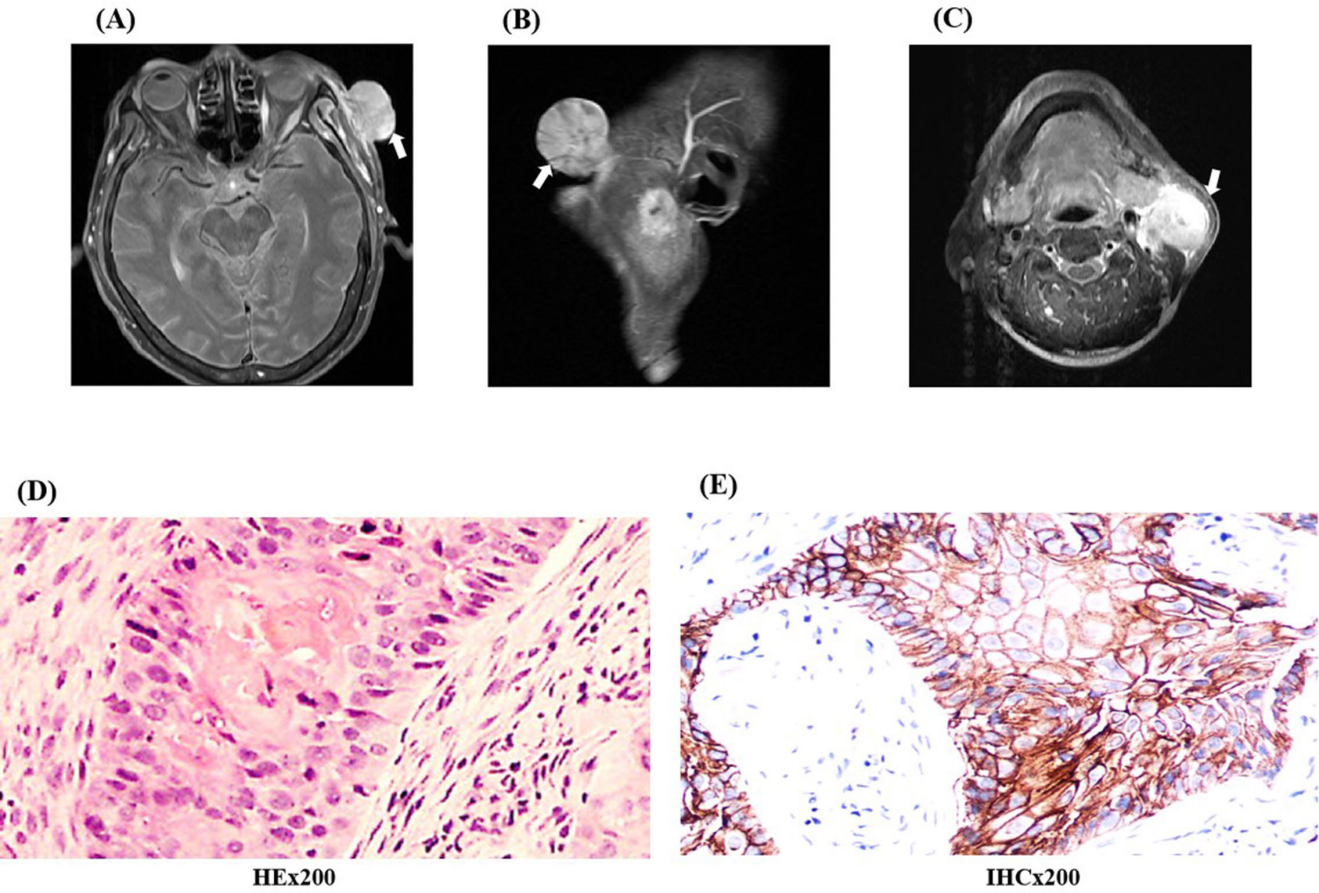
The pre-treatment images of the patient and pathology images. **(A–C)** Enhanced MRI images of the face and neck show an abnormally enhancing shadow in the left temporal region with a maximum diameter of 3.2 cm × 2.1 cm and enlarged left cervical lymph nodes. **(D)** The results of pre-treatment cervical lymph node aspiration biopsy suggest infiltration by highly differentiated squamous cell carcinoma. **(E)** Immunohistochemical findings of P63 (+) and PDL-1 expression. (HEx200, Hematoxylin and Eosin staining observed at 200x magnification; IHCx200, Immunohistochemistry staining observed at 200x magnification).

**Figure 2:**
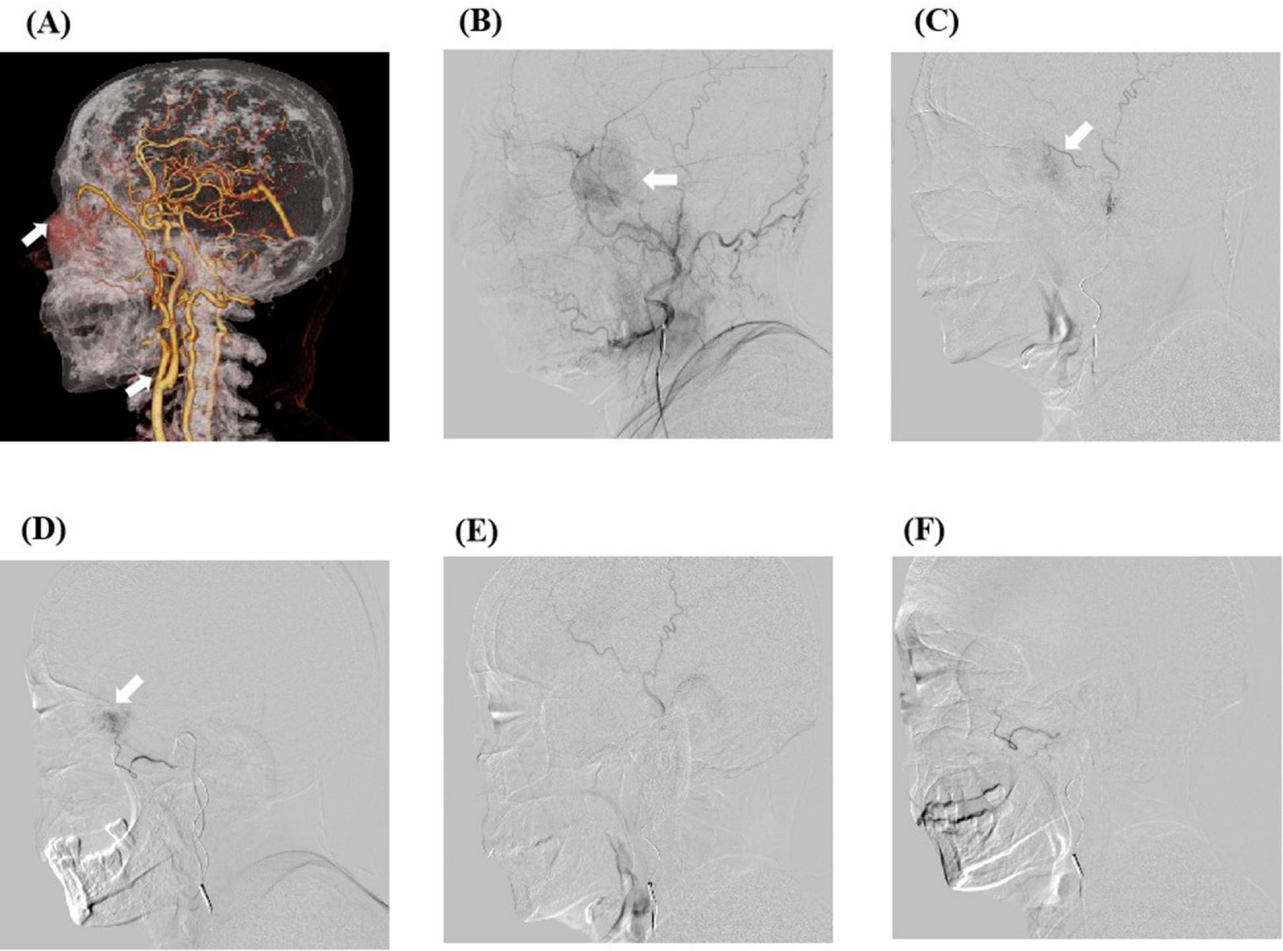
The preoperative arteriography results of the patient and the digital subtraction angiography (DSA) images of transcarotid chemoembolization. **(A)** The preoperative computed tomography angiography results of the patient show that the mass was supplied by the external carotid artery branch. **(B)** DSA image of the patient’s frontotemporal mass before embolization. After anesthesia, imaging examination through the left distal radial artery approach revealed an abnormal vessel with a diameter of about 3.5 cm on the left side of the face. A microcatheter was inserted into the external carotid artery branch, and 0.3 mg of gemcitabine was administered. The microspheres were then embolized with polyvinyl alcohol (blue spheres 100–300 microns in diameter) containing 0.5 g (1000 mg/m^2^) gemcitabine. **(C, D)** Intraoperative superselective arterial DSA images. **(E, F)** DSA images of the patient after embolization show that the tumor is not visible.

A 65-year-old female presented with a left frontotemporal mass had been admitted to our hospital on March 26, 2023. During the local examination, a mass was observed in the left frontotemporal region that measures approximately 4.5 cm × 4.5 cm × 3.0 cm, with black skin on the surface, a small amount of blood oozing, hard texture, easy to bleed on palpation, poor mobility, and pressure pain. In the systemic examination, one mass was palpable on the left side of the patient’s neck that measured approximately 2.0 cm × 2.0 cm × 1.0 cm, with a hard texture, poor mobility, no compression pain, and no skin abnormality on the surface of the mass. The Eastern Cooperative Oncology Group Performance Status(ECOG) of this patient is rated as 2, and she had a history of diabetes mellitus that had been occurring for several years. The admission enhanced magnetic resonance imaging (MRI) showed multiple mass-like abnormally enhanced shadows in the left temporal region, parotid area, below the parotid gland, and carotid sheath, with inhomogeneous enhancement (**Figures 1A–C**). The three-dimensional computed tomography examination of the blood vessels of the primary lesion suggested that the left frontotemporal lesion was supplied by the left superficial temporal artery, and the left submandibular and subparotid masses were supplied by the beginning segment of the external carotid artery (**Figure 2A**). In view of the fact that the primary tumor focus had a tendency to ulcerate and bleed and was prone to bleeding and concurrent infection after puncture biopsy, fine-needle aspiration biopsy of the cervical lymph nodes was selected instead. This approach was considered because it would result in a smaller wound and was safer. The results of pathological and immunohistochemical examination of neck mass puncture showed that highly differentiated squamous cell carcinoma infiltration was present in the fibrous tissue, with P63 (+) and PDL-1 expression (**Figures 1D, E**). Meanwhile, serum antigen detection was performed on the patient, which had certain reference significance for the diagnosis. The results indicated that the squamous cell carcinoma antigen level was 0.51 ng/mL and the carcinoembryonic antigen level was 1.17 ng/mL. After a multidisciplinary consultation, the patient was diagnosed with squamous cell carcinoma of the skin cTxN_1_M_0_.(The staging criteria are based on the 8th Edition of the American Joint Committee on Cancer.)

Due to the patient’s systemic conditions at that time and lesion characteristics, surgical treatment was not the optimal option.Her physical condition was poor, with an ECOG score of 2. Considering that she could not tolerate systemic treatment, and since the tumor was located in the head and neck area with relatively rich blood supply, DEB-TACE treatment was proposed.After communicating with the patient regarding the treatment plan and associated risks, she agreed to proceed with this treatment plan.

The patient underwent external carotid artery chemoembolization, which involves insertion of a microcatheter into the branch of the external carotid artery through the left radial artery, injection of gemcitabine 0.3 mg, followed by embolization with gemcitabine-loaded polyvinyl alcohol embolization microspheres in our interventional unit on April 3, 2023 (**Figures 2B–F**). On April 7, 2023, one cycle of tirilizumab (200 mg, once every 3 weeks) immunotherapy was carried out, and on April 11, 2023, the patient underwent gemcitabine combined with cisplatin local perfusion chemotherapy (gemcitabine 1000 mg/m^2^, cisplatin 75 mg/m^2^) via the left external carotid artery branch in the intervention room of our hospital. The examination results after this treatment cycle showed a decrease in white blood cell count, and the graded as Grade 2 according to the Common Terminology Criteria for Adverse Events (CTCAE Version 5.0). After symptomatic treatment of the elevated white blood cells, the blood cell counts returned to normal.

The patient was readmitted to our hospital on May 5, 2023. On examination, the left frontotemporal mass was significantly reduced compared with the previous findings, with a size of approximately 3.0 cm × 3.0 cm × 2.0 cm. The skin on the surface of the mass was mottled white, and there was mild pain on compression. The MRI results suggested that the cancerous foci were reduced to an extent compared with the previous observation. After admission to the hospital, the original program was continued for the second treatment cycle. Notably, while performing chemoembolization via the external carotid artery, we did not identify the blood-supplying arteries of the cancerous lesion. As the patient’s current physical condition has improved compared to before,she had an ECOG score of 1.We changed the treatment plan to gemcitabine (1000 mg/m^2^)single-agent intravenous chemotherapy. The test results of the treatment cycle showed that the white blood cell count was reduced, and the graded as Grade 2 according to CTCAE 5.0. The patient was then treated to relieve the drug-associated white blood cell elevation, and a follow-up blood test was performed. She was then followed up with two cycles of systemic chemotherapy (gemcitabine 1000 mg/m^2^ D1, D8, cisplatin 75 mg/m^2^) and tirilizumab (200 mg, every 3 weeks) immunotherapy at our department.

**Figure 3:**
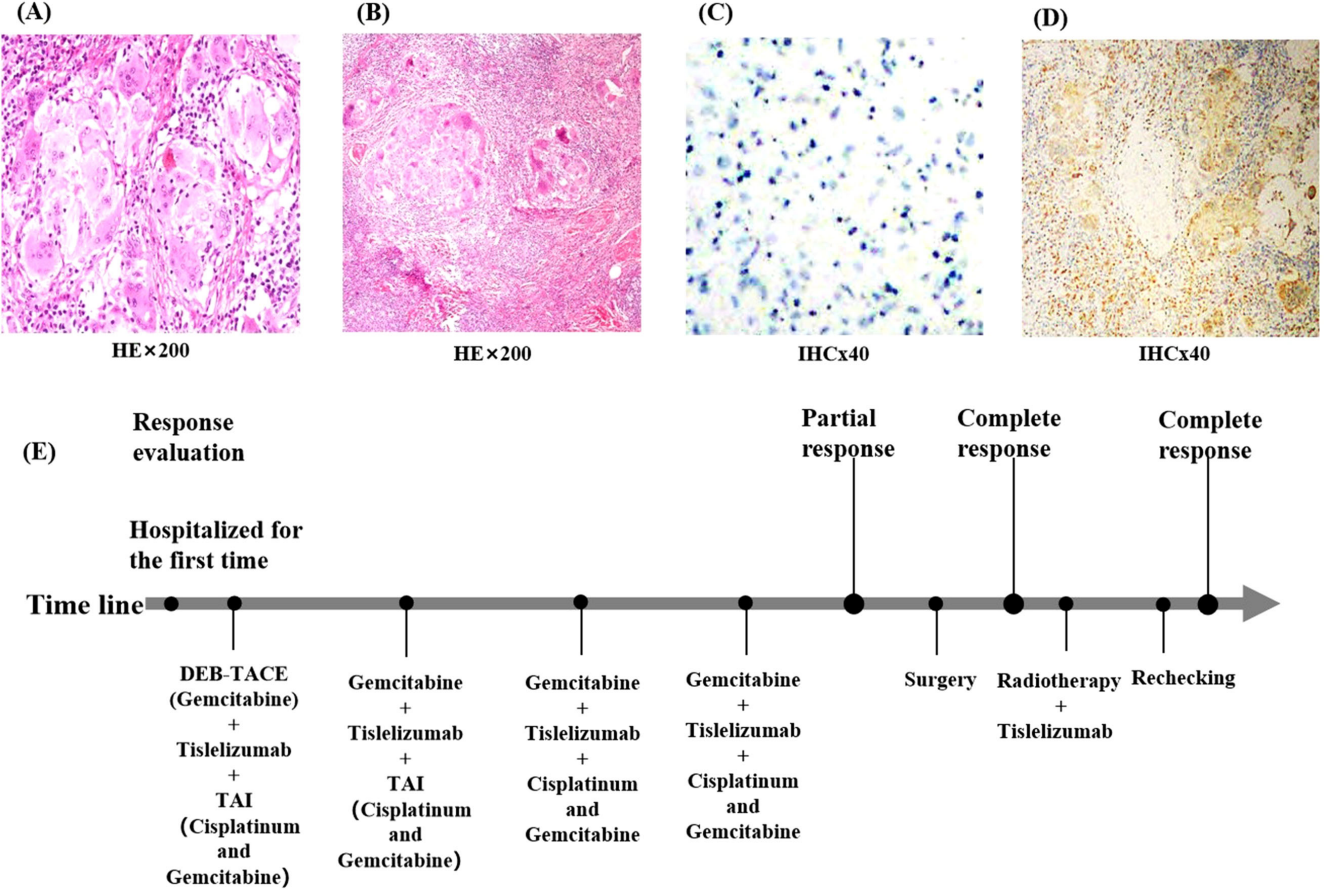
Pathological findings of the patient at the time of surgery. **(A)** Pathological findings of the facial mass suggest only squamous epithelial cell hyperplasia and no tumor cells. **(B)** Pathological results of the cervical lymph nodes suggest no active tumor cells were seen. **(C)** shows immunohistochemistry of the facial mass, suggesting P40 (−); **(D)** Postoperative immunohistochemistry of the cervical lymph nodes suggests CD68 (+). **(E)** Flow chart of the patient during treatment. (HEx200, Hematoxylin and Eosin staining observed at 200x magnification; IHCx40, Immunohistochemistry staining observed at 40x magnification).

The patient was then readmitted to our hospital. On examination, her left frontotemporal region only had postoperative crust residue, about 1 cm × 1 cm × 0.5 cm in size, and the surface skin of the swelling was mottled white without pressure pain. The MRI of her face and neck suggested that the left side of her face did not have any abnormal signal shadow.After multidisciplinary consultation, considering that the patient’s general condition was acceptable and the ECOG score was 0, it was recommended to proceed with surgery.Subsequently, the patient underwent left maxillofacial mass resection, left partial parotid gland resection, left cervical lymph node radical resection, and cosmetic skin closure in the ENT department of our hospital. No tumor cells were observed in the intraoperative frozen pathology of the left maxillofacial mass. The left cervical lymph node, parotid gland, and peripheral lymph nodes were collected and sent to the pathology department during the operation. The postoperative pathology and immunohistochemistry of the left maxillofacial mass revealed squamous epithelial cell hyperplasia, accompanied by hyperkeratosis and hyperkeratosis, and inflammatory exudates and necrotic material were seen in the epidermis. Multinucleated giant cell reaction with nodular distribution accompanied by fibroblastic tissue hyperplasia and calcification was seen in the dermis. No tumor cells were observed at the anterior margin of the mass, and cancer metastasis was seen in the lymph nodes sent for examination (1/13): 0/2 in the left parotid area, 1/5 in the left second area, and 0/6 in the left third area. Keratocyst: 1/5 in the left second region and 0/6 in the left third region. Immunohistochemistry of lymph node revealed CD68 (+), while immunohistochemistry of facial mass revealed P40 (−) (**Figures 3A–D**). P40 is a relatively specific squamous cell marker that can be expressed in squamous cell carcinoma. CD68 is a marker mainly used to label the mononuclear-macrophage cell system, which can identify the macrophages within the tumor microenvironment of the lymph nodes. By performing immunohistochemical detections of P40 and CD68 on the target lesions, it is possible to determine whether there are any residual tumor-associated antigens in the target lesions, providing a reference basis for subsequent treatment.The postoperative pathology of the patient showed no tumor cells on the face and no active cancer cells in the neck lymph nodes, but the CD68 expression in the neck lymph nodes was positive. This suggests that the patient’s facial lesions have completely disappeared and no antigen remains, although the cervical lymph node tumor cells are inactive, but there are still tumor associated antigens.Hence,the patient continued receiving postoperative radiation therapy combined with immunotherapy (tirilizumab 200 mg, every 3 weeks) for a period of 1 year. The radiotherapy plan is that for the area of positive lymph nodes, the total dose is set at 60 Gy, with irradiation administered once a day and five times a week. The treatment field encompasses a moderate outward margin of 1.5 centimeters added to the range of the left level II lymph node region.

**Figure 4:**
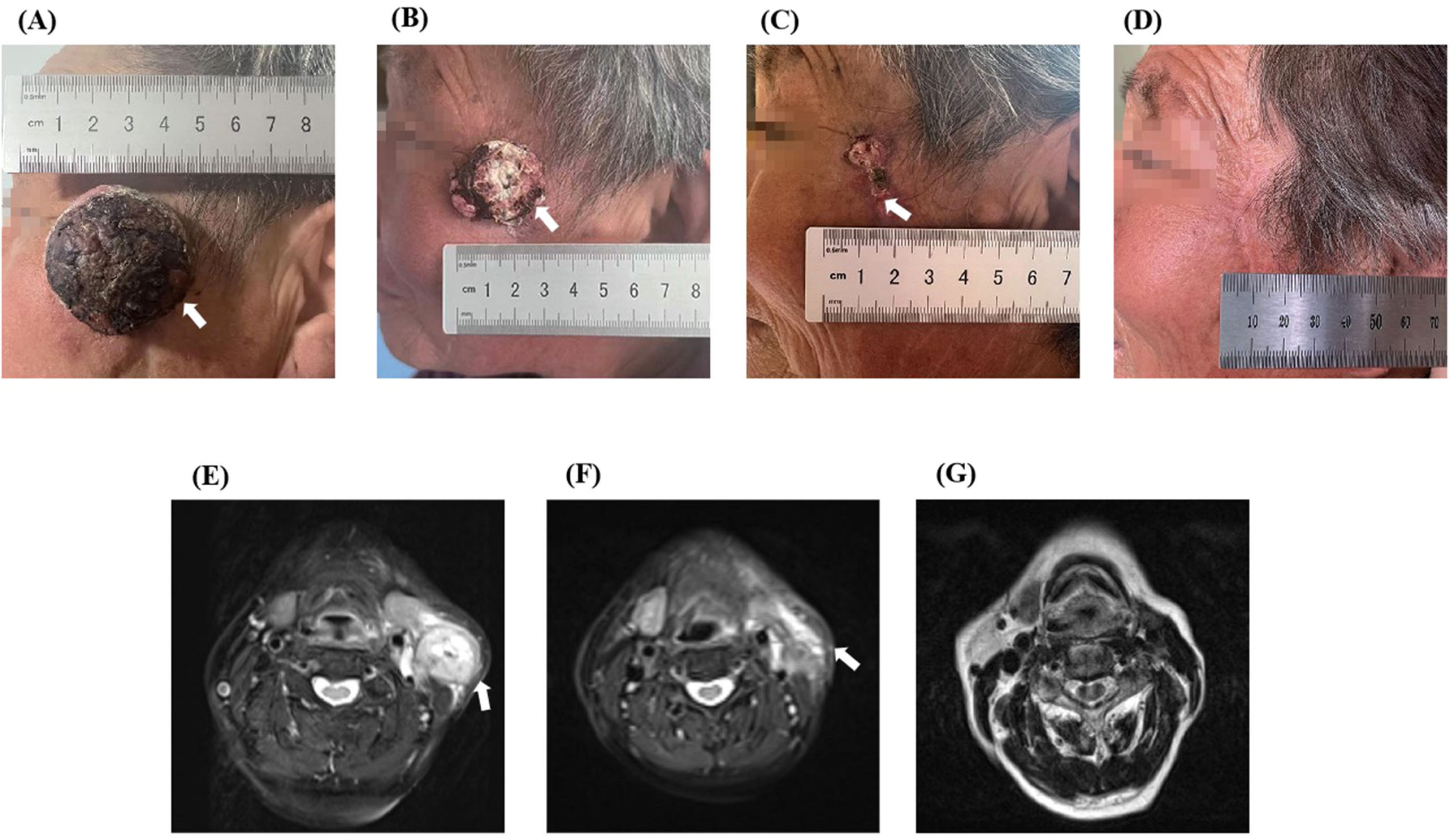
The comparison images of before and after treatment of the patient. Upon admission, a mass of about 3.5 cm × 3.5 cm was visible in the patient’s left temporal region, neck, and MRI showed abnormal signals in the shadow of the neck mass **(A, E)**. After one cycle of treatment, the quality was significantly lower than before treatment, and repeated MRI showed that the shadow quality of the neck was lower than before treatment **(B, F)**. **(C)** shows a visual view of the patient’s left temporal tumor after two cycles of treatment, leaving only postoperative scabs. **(D)** shows the visual observation of the patient after one year of treatment, leaving only postoperative scars, and **(G)** shows the MRI images of the patient’s neck after one year of treatment.

The patient is currently undergoing imaging every three months, and no signs of recurrence or metastasis have been detected. The last follow-up was on August 21, 2024. At the time of the recent follow-up visit, the patient had only postoperative scar tissue on her face (**Figure 4D**), and no abnormalities had been detected on nuclear magnetic plain scanning of the neck (**Figure 4G**). Thus, the patient presented no evidence of disease for 14 months. According to the Response Evaluation Criteria in Solid Tumors guidelines (version 1.1), the target lesion imaging was classified as a complete response, with a pathology suggestive of partial response (**Figures 4A–G**).

## 3 Discussion

cSCC is a malignant tumor caused by the uncontrolled proliferation of keratin-forming cells in the epidermis of the skin (5). It is the second most common non-melanoma skin cancer, accounts for approximately 20% of all skin cancers, and is increasing in incidences annually, with a great impact on human life (6). The occurrence of cSCC has been associated with environmental factors, age factors, β-human tumor virus subtype infection, smoking, immune factors, genetic factors, and other factors. The early stages of cSCC often present as hard papules or erythematous nodules with distinctive scales that often have a tendency to bleed. The definitive diagnosis of cSCC includes histopathological biopsy, dermoscopy, and reflection confocal microscopy, which is the gold standard for diagnosing the disease. Common treatment options for cSCC include surgical treatment, local nonsurgical treatment, radiation therapy, and systemic therapy (7). Surgical resection is the preferred option for cSCC; however, the standards for surgical resection are high to ensure negative margins. Furthermore, the procedure often results in a traumatized surgical area that is prone to complications such as hemorrhage, nerve damage, and infection (8). Local nonsurgical treatments, including cryotherapy, laser, and photodynamic therapies, are only suitable for cSCC with a small diameter and low risk (5). Radiation therapy has some value in the treatment of cSCC, but its drawbacks include a lower cure rate and a tendency for recurrence; therefore, it is usually used as an adjunctive treatment for cSCC in advanced stages or with local tissue invasion (8). Systemic drug therapies can be administered as the disease progresses. Prior studies have pointed out that platinum-based chemotherapy, gemcitabine, ICI (cimeplizumab), and epidermal growth factor inhibitors (cetuximab) have a certain degree of efficacy in the treatment of late-stage cSCC (1, 9). Some scholars have proposed that multidrug combination therapy may be more effective than single-drug treatment; however, there is a lack of evidence-based support in China and abroad (10). As cSCC is common in older populations, cytotoxic drugs have more toxic side effects, such as myelosuppression and abnormal cardiac function, which may affect patient survival.

With the development of medical imaging technology, interventional therapy has gradually entered the public’s field of vision, with the advantages of high efficiency, safety, targeting, accuracy, fewer adverse reactions, and being highly praised by most doctors and patients. Known interventional therapies include intra-arterial therapy (such as transarterial embolization, transarterial chemoembolization, transarterial perfusion chemotherapy, and drug-eluting bead arterial chemoembolization), ablation, brachytherapy, and other modalities (11). DEB-TACE is often used to treat liver and lung cancers. And Bi et al. (4) pointed out that DEB-TACE is safe and effective for treating patients with advanced or recurrent head and neck cancer. In this study, for the patients treated with DEB-TACE, the objective response rate and disease control rate at 6 months after the operation were 25% and 69%, respectively. The median overall survival and local progression-free survival were 14.5 months and 13.6 months, respectively. However, when DEB-TACE is used for the treatment of head and neck malignancies, there is a risk that it may lead to embolism in the head and neck arteries, which could subsequently trigger cerebral infarction. During the operation, the target vessels must be super-precisely selected before embolization can be carried out. If a significant slowdown in blood flow is detected during the operation, the surgical procedure must be stopped immediately to prevent the occurrence of adverse consequences (12). Throughout the entire operation process, strict measures should be taken to prevent the backflow of embolic materials to ensure the life and health safety of patients. During the DBE-TACE treatment, the patient only experienced a decrease in white blood cells. After treatment to increase white blood cell count, the blood cell count recovered, and no serious adverse reactions such as cerebral infarction occurred.DEB-TACE was chosen as the treatment method, which is based on the principle that microspheres containing cytotoxic drugs are used to embolize the arteries that supply blood to the tumor and release cytotoxic drugs while simultaneously blocking the arteries that supply blood to the tumor. The target lesion is exposed to a high concentration of the drugs, which reduces the load of the tumor in the short term, thus efficiently eliminating tumor necrosis, relieving clinical symptoms, and improving therapeutic response. This method can more effectively eliminate tumor necrosis, thus relieving clinical symptoms and improving therapeutic response. Gemcitabine combined with cisplatin is usually used as the first-line treatment option for patients with advanced squamous non-small cell lung cancer, and it has been shown that gemcitabine-loaded arterial chemoembolization in the treatment of advanced or inoperable lung cancer can significantly improve patients’ overall quality of life and physical function (3, 13).

Currently, immunotherapy is becoming a new trend in treating malignant tumors, and the application of ICI in treating advanced cancers provides new hope for patients. Studies have shown that the tumor necrosis rate is low when tumors are treated with transarterial chemoembolization alone, and it is difficult to achieve the desired effect with this method alone. However, combining chemoembolization with ICI improves therapeutic efficacy, and the two modalities present a synergistic effect (14). Tirilizumab is a monoclonal antibody with high affinity and specificity for programmed death 1, which is specifically designed to minimize the binding of Fcγ receptors to macrophages, thus eliminating antibody-dependent phagocytosis. Furthermore, it has been shown to be effective in the treatment of squamous cell carcinoma (15).

In this patient, because the cancer lesion was located in the frontal face, the anatomy of the surrounding tissues was complicated. The diameter of the cancer lesion was large, and the patient had a combination of diabetes mellitus and other chronic diseases. The surgical treatment was traumatic, with more postoperative complications and poorer therapeutic efficacy. Furthermore, it was found that the lesion was supplied by a branch of the external carotid artery during the preoperative checkup, which was in line with the indications of interventional therapy, so we chose to treat the patient with interventional therapy combined with immunotherapy. Transcarotid artery perfusion chemotherapy (cisplatin combined with gemcitabine), immunotherapy (tirilizumab), and DEB-TACE (gemcitabine) were applied sequentially during the treatment cycle. The patient received four cycles of treatment (**Figure 3E**), and the tumor shrank progressively, the color changed from black to mottled white, the pressure and pain of the lesion gradually decreased, the lymph nodes in the neck shrunk to 1.0 cm, and no tumor cells were identified in the frontal and facial crusts at the time of the surgical procedure. The patient was re-examined one year later and presented only residual surgical scarring on her face and no abnormalities in the neck MRI. The response of the target lesion was classified as a complete response according to Response Evaluation Criteria in Solid Tumors guidelines (version 1.1), and the pathological response was a partial response. No recurrent metastasis of the lesion was observed during the follow-up period, and patient satisfaction was high. Interventional therapy provides a novel option for patients with squamous cell carcinoma of the skin, and this study may provide a reference for clinicians when deciding on the most appropriate treatment option for their patients.

## 4 Conclusion

We combined DEB-TACE with ICI to treat the patient with SCC. After treatment, her target lesions achieved a complete response, and the pathological examination showed a partial response. In addition, no signs of recurrence were detected during the follow-up, and the patient remained disease-free within 14 months. During the treatment process, the patient only experienced a decrease in white blood cells, and after treatment to increase the white blood cell count, the blood cell count returned to normal. No serious adverse reactions such as cerebral infarction occurred. This treatment regimen demonstrated good safety and efficacy in this case. In clinical practice, for malignant tumors in special locations, if the patient’s physical condition cannot tolerate systemic treatment or surgical treatment, we can choose DEB-TACE based on the blood supply of the tumor site to reduce the adverse reactions of the drugs. However, more large-scale clinical studies are still needed in the future to further validate the above conclusions.

## Data Availability

The original contributions presented in the study are included in the article/supplementary material. Further inquiries can be directed to the corresponding author/s.
